# Effects and Mechanisms of Silicone Fertilizer on Salt Ion Activity in Saline–Alkaline Soils

**DOI:** 10.3390/polym18020231

**Published:** 2026-01-16

**Authors:** Furu Song, Dongxia Li, Liqiang Song, Ziku Cao, Zhipei Cao, Yafei Sang, Lianwei Kang

**Affiliations:** 1 Hebei Silicon Valley Fertilizer Co., Ltd., Handan 056038, China; 2 School of Water Conservancy and Hydropower, Hebei University of Engineering, Handan 056038, China; 3 Soil and Fertilizer Station of the Agricultural and Rural Affairs Bureau of Yongnian District, Handan 056038, China; 4 Hebei Silicon Valley Agricultural Science Research Institute, Handan 056038, China; 5 College of Materials Science and Engineering, Hebei University of Engineering, Handan 056038, China

**Keywords:** organosilicon fertilizer, saline–alkali soil, salt ion concentration, Lewis acid-base action

## Abstract

The high salt content in saline–alkali soil has a significant impact on plant nutrient absorption and water transport, severely inhibiting crop growth. Through esterification reactions, silicic acid is grafted onto humic acid to form an organic silicon fertilizer (OSiF). The unique Si-O-C bond in the material endows this new type of organic silicon-based fertilizer with the ability to effectively alleviate the harm of high-salt soil to plants. In this study, a soil column experiment was designed to systematically evaluate and compare the effects of organic silicon fertilizers with different organic silicon contents (0%, 5%, and 10%) and traditional compound fertilizers on soil water characteristics, salt ion concentration, pH value, and electrical conductivity. The results showed that the addition of an appropriate amount of organic silicon fertilizer could significantly reduce the activity of salt ions in the soil solution. Experimental data indicated that the 5% and 10% organic silicon fertilizers had the most significant effect on the consumption of major salt ions such as sodium and chloride ions. X-ray photoelectron spectroscopy (XPS) analysis revealed that the reaction of Si-O-C bonds in the soil with Lewis bases led to a shift in the valence state of the 1S electrons of silicon atoms, providing a theoretical basis for the mechanism by which silicon fertilizers alleviate high-salt stress.

## 1. Introduction

The Songnen Plain is one of the world’s three major saline–alkali soil regions. Salinization is a significant factor restricting regional economic development and improving the ecological environment. The damage caused by saline–alkali land in the Songnen Plain to the agricultural ecosystem is typical and severe. When the soil pH value exceeds the critical threshold of 7.3, it forms strongly alkaline–saline soil. This special soil environment poses multiple hazards to agricultural production [1,2,3,4,5,6].

According to soil classification standards, when the soil pH value exceeds 7.3, it is defined as alkaline soil. Such soil typically shows an obvious accumulation of soluble salts and a lack of organic matter, which leads to a decrease in cation exchange capacity, a reduction in aggregate stability, and a decline in permeability [7,8,9,10]. This structural degradation leads to an increase in soil bulk density, a decrease in total porosity, and thus a lack of oxygen in the soil. In this oxygen-deficient environment, the aerobic respiration metabolism of crop roots is hindered [11,12,13]. The inhibition effect of salt stress on crop growth and development is significantly concentration-dependent. Qi Tong et al. [14] showed that when the salt concentration exceeded 9‰, the growth rate of corn germ was significantly inhibited. It is worth noting that Zhao et al. [15] discovered that low salt concentrations have a positive regulatory effect on solanaceous crops, indicating that salt stress has a typical “low promotion, high inhibition” dual-phase regulation on plant growth. This concentration-dependent effect may be closely related to the ion homeostasis regulation mechanism of plants, the dynamic response of the osmotic balance system, and the antioxidant defense system. Silicon (Si) is the second most abundant element in the Earth’s crust, mainly existing in the form of silicates and silicon dioxide. Although silicates make up 87% of the composition of the terrestrial lithosphere, the available silicon content that plants can absorb is usually low because most of them exist in crystalline form or combined with metal oxides [16,17,18]. Recent studies have shown that silicon, as a beneficial element for plant growth, significantly enhances the mechanical strength of vascular bundles by mediating the process of cell wall silicification, promotes root morphological development, and increases the vertical growth of stems and leaves to improve photosynthesis [19,20,21,22]. The use of chemical methods to improve saline–alkali soil is currently a research hotspot. Chemical improvement measures mainly involve the application of chemical amendments such as humic acid and high-molecular-weight polymers, which react with salt ions in the soil to improve soil structure. Cheng et al. [23] analyzed the improvement effect of desulfurized gypsum on the coastal saline–alkali soil in Dongtan, Shanghai, through indoor soil column leaching experiments. The research found that adding desulfurized gypsum could increase the porosity of the saline–alkali soil, significantly enhance the saturated hydraulic conductivity of the soil, and accelerate the soil desalination process. The research results of Zhang et al. [24] showed that adding desulfurization gypsum can reduce the proportion of soil clay particles, improve soil physical structure, increase soil porosity and permeability, and effectively accelerate the process of leaching desalination. Sun et al. [25] conducted an indoor simulation experiment, which demonstrated that after the addition of aluminum sulfate, the sodium ions, carbonate ions, and bicarbonate ions in the soil solution significantly increased, and the pH value of the soil solution decreased significantly. A study by Wang et al. [26] compared the effects of two amendments (PAM and Dryland Dragon) on the infiltration characteristics of sandy saline –alkali soil. The results showed that the application of both materials effectively reduced soil moisture loss. When the application rates of PAM and Dryland Dragon were 0.04% and 150 g·m^−2^, respectively, the soil layer had the best water holding effect and the total amount of salt leaching was the highest. Sun et al. [27] improved the saline–alkali soil in the Yellow River Delta by adding furfural residue. The results showed that the soil pH value decreased, and the addition of 5% furfural residue increased the available phosphorus content in the soil by 4–6 times while significantly reducing the soil alkalinity. Yao et al. [28] improved the soda saline–alkali soil in Jinzhong, Shanxi Province, by mixing fly ash and vinegar residue. The research results showed that the soil bulk density decreased after the improvement, the soil permeability increased, and the content of available potassium in the soil significantly increased. This series of studies systematically reveals how saline–alkali land impairs crop growth and validates two effective improvement strategies: the application of silicon and the use of chemical modifiers (such as desulfurized gypsum).

This study focuses on the salinization problem of soda saline–alkali soil in the western part of the Songnen Plain and reveals the key mechanism of organic silicon fertilizer (OSiFs) in the improvement of saline–alkali soil through multi-scale experiments. The experiments found that when 5–10% of organic silicon fertilizer was applied, the activity of basic ions such as Na^+^ and Cl^−^ in the soil solution was significantly reduced, and the soil pH value also decreased significantly. Characterization by X-ray photoelectron spectroscopy (XPS) revealed that the Si-O groups in the organic silicon molecules formed coordination bonds with the soil Lewis bases, resulting in a change in the binding energy of the Si 1s electrons. This chemical bond effectively blocked the migration activity of salt ions. The combined application of organic silicon fertilizer and traditional inorganic silicon fertilizer can reduce the electrical conductivity of the crop rhizosphere, providing a theoretical basis for solving the problem of ion toxicity in high-saline–alkali soil.

## 2. Experimental Methods

### 2.1. Materials

**Figure 1:**
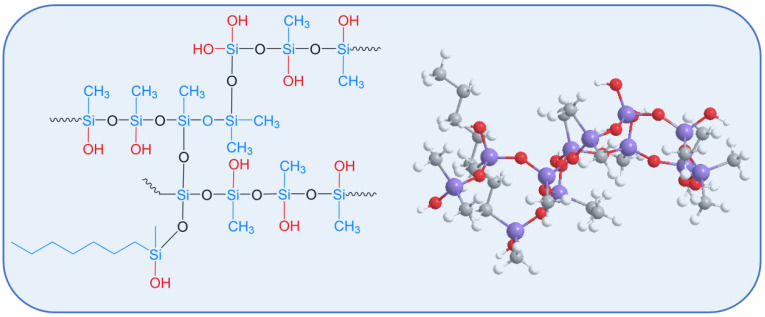
Molecular structure fragments of modified organosilicon.

The soil was collected from 0 to 20 cm of surface soil in the 10,000 mu demonstration field of Hebei Silicon Valley Fertilizer Co., Ltd., Longmian City, China. The soil characteristics belong to the typical soda saline–alkali soil in Northeast China. OSiF, provided by theHebei Silicon Valley Academy of Agricultural Sciences, is made by coating a silicomethylated resin polymer on a particle-mixed composite fertilizer containing N, P, and K nutrients, with the content of N, P, and K being 18-18-18 (that is, N, P_2_O_5_, and K_2_O account for 18% of the total mass of the fertilizer, respectively). OSiF is a completely water-soluble fertilizer with slow-release properties. The polymethylsiloxane-modified organosilicon was furnished by the Hebei Silicon Valley Academy of Agricultural Sciences. It was generated through the hydrolysis and condensation reaction of dichlorodimethylsilane, trichloromethylsilane, triethoxymethylchlorosilane, and Cn H_2n+1_(CH_3_)Si(OCH_3_)_2_ to form a methylsilicon resin. Subsequently, under strongly alkaline conditions and after catalytic hydrolysis modification, some Si-O-Si bonds were hydrolyzed and broken, generating Si-OH groups. The modified organosilicon obtained through hydrolysis possesses a multi-branched molecular structure that integrates both hydrophobic -CH_3_ and hydrophilic -OH groups. Its structural formula is depicted in Figure 1. Aluminum trichloride, copper sulfate, zinc chloride, and sodium bicarbonate, from Shanghai Aladdin Biochemical Technology Co., Ltd. (Shanghai, China), are pure analytical reagents.

### 2.2. Experimental Design

**Figure 2:**
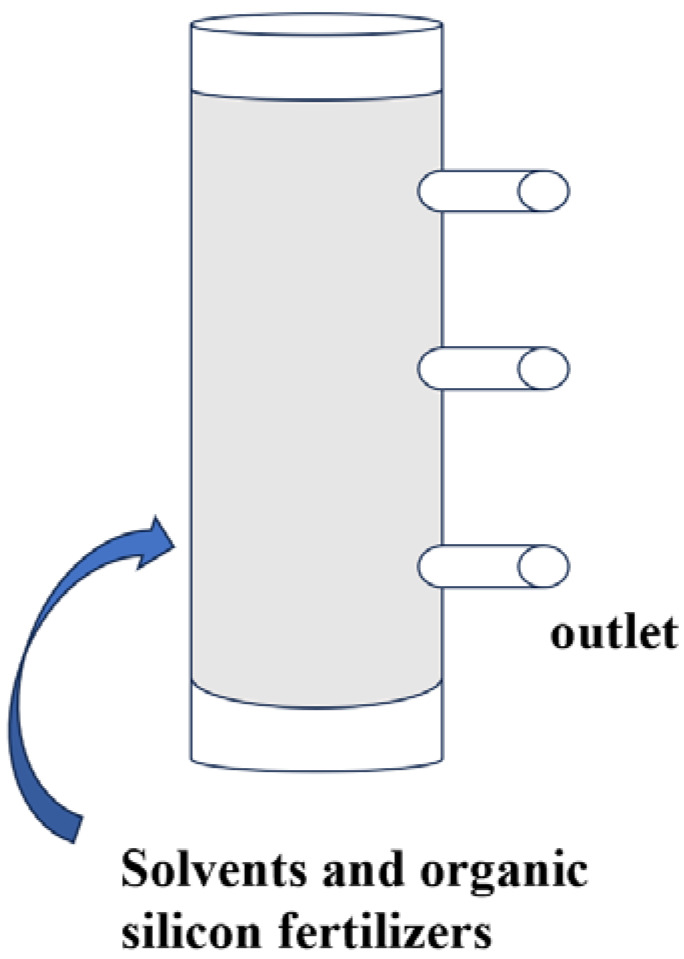
Soil column experimental apparatus.

The indoor one-dimensional vertical homogeneous soil column infiltration experiment was performed as follows. The soil was naturally air-dried, and impurities like stones were removed, ground, and passed through a 2 mm sieve. The soil column was filled with five layers, with 337 g for each layer, and the soil bulk density was 1.325 g/cm^3^. The soil column was a transparent resin tube with an inner diameter of 9 cm and a height of 40 cm. A fixed water head of 8 cm was adopted to supply water to the soil column. The experiment ended when the water volume corresponding to a water head of 8 cm was reached [2,4]. After the infiltration, soil samples were taken from the soil column’s reserved positions (the top, middle, and bottom layers), as shown in Figure 2 below.

Three treatments were set up in the experiment: saline–alkali soil (CK-Y), saline–alkali soil + 5% silicon fertilizer (Y+F), and saline–alkali soil + 10% silicon fertilizer (Y+5%). Three parallel experiments were conducted for each treatment. Five kilograms of silicon fertilizer per mu was considered as 5%. The treatments included surface soil (CK), surface soil + compound fertilizer (G+F), surface soil + 5% organic silicon fertilizer (G+5%), surface soil + 10% organic silicon fertilizer (G+10%), saline–alkali soil (Y), saline–alkali soil + compound fertilizer (Y+F), saline–alkali soil + 5% organic silicon fertilizer (Y+5%), and saline–alkali soil + 10% organic silicon fertilizer (Y+10%). Deionized water and saline water climbing was performed as follows: the soil column was divided into six layers, where each layer was 3 cm. The soil bulk density was 1.325 g/cm^3^, and the inner diameter of the soil column was 5 cm. Each layer was filled with 78 g of soil, and the water flow was controlled using a Marsh bottle, Chongqing xinweier Glass Co., Ltd., Chongqing, China. 

The soil sample analysis was performed as follows: the collected soil sample was divided into two parts. One part of the soil sample was dried in the oven to determine the moisture content of the soil after infiltration; the other part of the soil sample was naturally air-dried to prepare the soil extract, which was used to determine the soil conductivity and pH [8,9].

### 2.3. Preparation of Soil Extracts

According to the literature before the experiment, most soil leaching solutions are prepared according to the following ratios of soil to water: 1:1, 2:1, 5:1, and 10:1. In this paper, soil leaching solutions were prepared according to a ratio of soil to water of 5:1. An analytical balance was used to accurately weigh 20 g air-dried soil into a beaker; then, 100 mL of deionized water was added. The solution was stirred, pumped, and filtered, and the filtrate was stored in a 100 mL volumetric bottle; that is, a 5:1 soil leaching solution [8].

### 2.4. Determination of Total Salt Content

The determination of total salt content in soil is mainly by the gravimetric method. A 10 mL pipette was used to measure 10 mL of the soil leach liquid into a beaker with constant weight and put in an oven at 105–110 °C for drying, cooling, weighing, and calculating the total salt content [14].

### 2.5. Determination of Soil pH and Conductivity (Ec) Values

An amount of 30 mL of soil extract was measured in a small beaker; the pH value was determined with a Mettler pH meter, Mettler Toledo group, Shanghai, China, and the electrical conductivity was determined with a Thunder magnetic conductivity meter [15].

### 2.6. Characterization

X-ray photoelectron spectroscopy (XPS) was collected using Escalab250xi Thermo Fisher Scientific, Waltham, MA, USA with a monochromatized Al Ka X-ray source. The crystal structure of the polymers was characterized by X-ray diffraction (XRD) on a Rint-2000 diffractometer, Japan Science Corporation, Tokyo, Japan with Cu Kα radiation. ^1^H and ^13^C nuclear magnetic resonance (NMR) spectra were obtained on a Bruker Avance III, Brooke Corporation, Billica, MA, USA with a magnetic field strength of 11.7 T. Fourier transform infrared (FT-IR) spectra of the polymers were recorded by a Nicolet 6700 (Thermo Scientific Co., Waltham, MA, USA). A Leica inverted microscope was used for observing the morphology of samples.

## 3. Results and Discussion

### 3.1. Infiltration and Ascent of Soil Moisture

**Figure 3:**
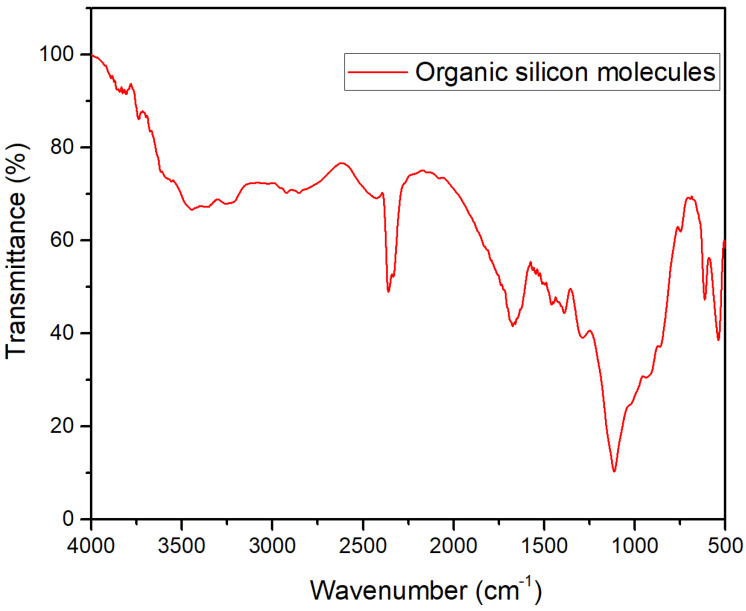
FT-IR spectra of organic silicon molecules.

Infrared analysis was conducted on the molecular structure of organosilicon, and the results are shown in Figure 3. In the infrared spectrum, the characteristic peak at 700–860 cm^−1^ is the absorption peak of Si-C. The characteristic absorption peak of Si-O-Si is located at 1000–1200 cm^−1^ [29,30], and the absorption peaks near 3450 cm^−1^ and 1600 cm^−1^ correspond to the absorption peak of -OH [31]. This indicates that organic silicon molecules mainly have a chain structure composed of Si-O and Si-C bonds, confirming the proposed organic silicon synthesis structure. Combined with the synthesis monomers of organic silicon molecules, it can be found that organic silicon molecules mainly have a main chain structure of Si-O-Si chains and a multi-group macromolecular structure composed of Si-C bonds. The characterization of organic silicon molecular products using H-NMR is shown in Figure S1. The peak between 0 and 2 ppm in the nuclear magnetic spectrum is the characteristic peak of CH_3_, corresponding to the Si-CH_3_ group in the organic silicon molecule product and the methyl structure in the side chain [32]. The results are consistent with the results of the infrared analysis.

Due to the high molecular weight of the synthesized organosilicon molecules, Py-GCMS testing technology was further used to detect the organosilicon molecules (Figure S2). It can be observed that after pyrolysis, the organic silicon molecules are decomposed into small molecular fragments. Through GC-MS analysis and comparison with the database, the main structure of the organic silicon molecules is found to be a linear structure with Si-O-Si as the main chain, which intuitively proves the structure of the organic silicon molecules and is consistent with the speculated molecular structure and the analyzed structure mentioned above [33].

The stability of organic silicon molecules was studied through thermogravimetric analysis. As shown in Figure S3, the molecule exhibits its first maximum weight loss peak at approximately 60 °C, mainly due to the loss of water in the sample during the heating process. Starting from 100 °C, the second weight loss occurred, and the second maximum weight loss peak appeared at about 174 °C, mainly due to the decomposition of oxygen-containing functional groups, -OH, indicating that organic silicon molecules have good thermal stability. At 800 °C, approximately 72% of the residue remained, mainly consisting of Si-containing substances, indicating a high content of Si element in the synthesized organic silicon molecular products. Silicon is an important component of soil and plays a key role in regulating soil properties. The above results fully demonstrate the structural characteristics of the synthesized organosilicon molecules. Due to the abundant and alternating hydrophobic -CH_3_ and hydrophilic -OH groups on their main chain and branches, they are endowed with various special functions such as water absorption and retention, ion complexation, etc. In addition, their good thermal stability makes mixing with other base fertilizers easier.

**Figure 4:**
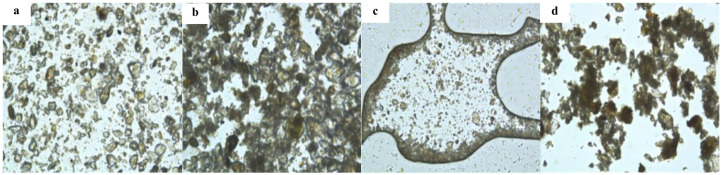
(**a**) Saline–alkali soil; (**b**) saline–alkali soil silicon fertilizer; (**c**) clay; (**d**) clay silicon fertilizer.

The dispersion forms of different soils in water were analyzed. Pure soil and soil with added organic silicon compound fertilizer were placed in pure water and observed using a Leica inverted microscope. The results are shown in Figure 4. Through comparison, it was found that pure saline–alkali soil can be uniformly dispersed in water, mainly due to the higher ion concentration in saline–alkali soil, which can be well dispersed in water by water solvation. After adding organic silicon compound fertilizer, soil particles aggregate into large particles, indicating that organic silicon compound fertilizer can promote particle aggregation in saline–alkali soil. Similarly, clay samples were dispersed after being placed in water, and the addition of organic silicon water-soluble fertilizer resulted in significant soil agglomeration. This indicates that organic silicon compound fertilizer can effectively promote soil granulation, mainly due to the presence of Si-O-Si bonds and free hydroxyl groups in organic silicon molecules in organic silicon water-soluble fertilizers, which can interact with saline–alkali soils [34,35]. The ions in the soil form coordination bonds and hydrogen bonds, reducing their concentration in the soil. On the other hand, Si-O-Si bonds and hydroxyl groups at different positions adsorb soil particles, causing multiple soil particles to “stick” together.

### 3.2. Soil Moisture Climbing

**Figure 5:**
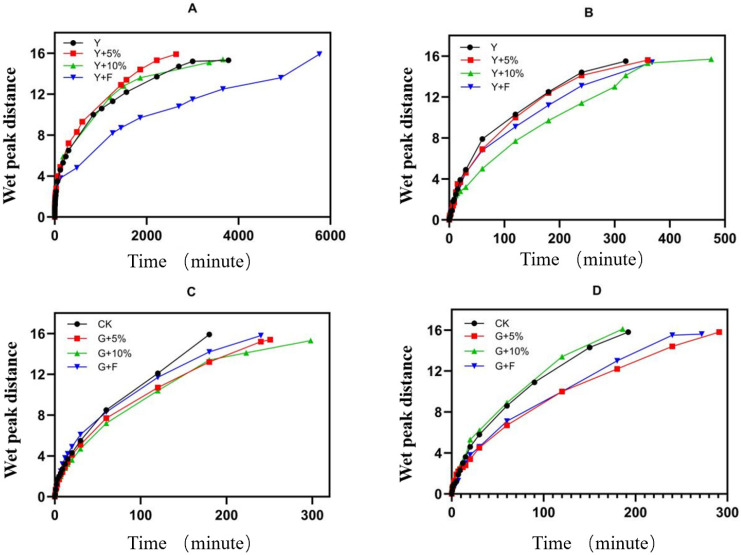
Comparison of rising groundwater levels between saline–alkali soil and cultivated soil: (**A**) salt–alkali soil and deionized water rise; (**B**) saline–alkali soil and saline water rise; (**C**) deionized water rise in the topsoil layer; (**D**) the saline water in the topsoil is rising.

Figure 5 shows a comparison of the rise in groundwater between saline–alkali soil and cultivated soil. The characteristic curve of soil moisture rise indicates that organic silicon has a significant promoting effect on the rise in deionized water in saline–alkali soil and a significant slowing effect on the rise in saline–alkali soil. Moreover, the rise in moisture is correlated with the amount of organic silicon used. Organic silicon has a slowing effect on the rise in deionized water in topsoil, and 5% organic silicon compound fertilizer has a significant slowing effect on the rise in saline water in topsoil.

### 3.3. Determination of Total Salt Content, Conductivity, and pH of Soil at Different Depths

The effect of adding organic silicon fertilizer on soil alkalinity was determined. An amount of 20 g of air-dried soil was accurately weight and placed in a beaker. Then, 100 mL of deionized water was added and stirred thoroughly, and suction filtration was conducted. The filtrate was then volumetrically fixed to 100 mL in a volumetric flask, constituting a 5:1 soil leachate. An amount of 30 mL of the soil leachate was measured in a small beaker, and the pH value was determined using a Mettler pH meter. Electrical conductivity was measured using a Leici conductivity meter. With a 10 mL pipette, 10 mL of the soil leachate was measured and transferred to a beaker that was dried to a constant weight. The beaker was placed in an oven at 105–110 °C for drying. It was cooled down and weighted, and the total salt content was calculated. The experimental results are presented in the table below. It can be seen from the experimental results that after adding organic silicon fertilizer, the salt content of water in the middle and lower layers of the column experiment increases, the conductivity of water increases, and the pH value of soil becomes weak, which proves that the application of organic silicon fertilizer can effectively reduce the alkalinity of soil (Table 1).

### 3.4. Interaction Between Organosilicon Molecules and Metals

**Figure 6:**
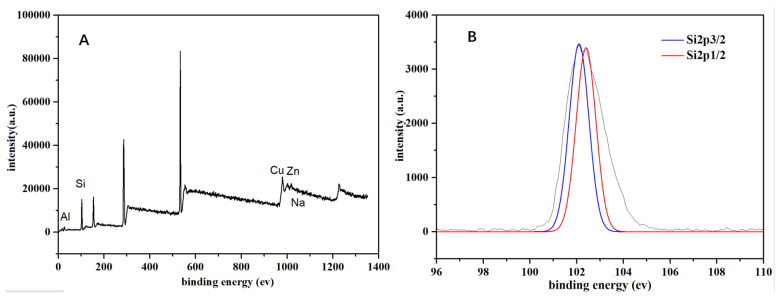

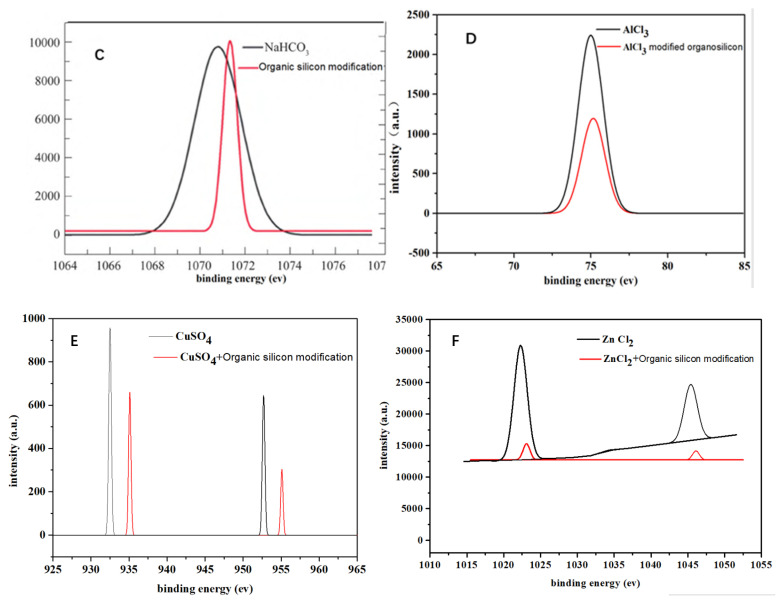
XPS spectra of modified silicone and characteristic XPS spectra of metal ions before and after reaction with different metal ions. ((**A**) is modified organosilicon; (**B**) is Si; (**C**) is Na; (**D**) is Al; (**E**) is Cu; (**F**) is Zn).

Organosilicon molecules will interact with metal oxides in saline–alkali soil through Lewis acid–base interactions. X-ray photoelectron spectroscopy (XPS) is used to analyze the elemental composition of organosilicon molecules and their interactions with Lewis acids. The full spectra of the modified organosilicon molecules are shown in Figure 6A. Peaks such as O1s, Si2p, and C1s can be observed. The binding energy of O1s is 532.74 eV, and the bond is a Si-O bond. Si2p includes two main peaks, Si2p3/2 and Si2p1/2, as shown in Figure 6B, indicating that there are two chemical forms of Si in the modified organosilicon molecules. The binding energy of Si2p3/2 is 102.1 eV, and the bond is a Si-C bond, while the binding energy of Si2p1/2 is 102.4 eV, and the bond is a Si-O bond [36]. The results after the interaction of the modified organosilicon molecules with Lewis acids are shown in Figure 6C–F. Compared with before the reaction, the binding energies of Al2p, Zn2p, Cu2p, and Na1s electrons have shifted in the direction of higher binding energy to varying degrees, and the O atom in the organosilicon molecules acts as an electron-withdrawing group, with an electronegativity of 3.44. Therefore, the attraction of the O atom in the organosilicon molecules to the outer electrons of the metal atoms is stronger, resulting in a decrease in the density of the outer electrons of the metal atoms and a weakening of the electron shielding effect and an increase in the binding energy of the inner electrons. Thus, the binding energies of Al2p, Zn2p, and Cu2p electrons of these metals shift in the direction of higher binding energy to varying degrees [37,38,39]. Meanwhile, after the O atom in the modified organosilicon molecules attracts electrons, the outer electron density increases, and the electron binding energy of O1s decreases to varying degrees (Table S1). This indicates that CuSO_4_, ZnCl_2_, AlCl_3_, and NaHCO_3_ have interacted with the organosilicon molecules to varying degrees. Thus, it is confirmed that there are indeed different degrees of interaction forces between the modified organosilicon molecules and Lewis acids and proton bases.

## 4. Conclusions

This study systematically evaluated and comparatively analyzed the effects of different proportions of organic silicon fertilizers (OSiFs) (0%, 5%, and 10%) on the dynamic characteristics of soil moisture, the distribution of soluble base cations, pH buffering performance, and the evolution laws of electrical conductivity. The experimental data indicated that the application of appropriate amounts of OSiFs could significantly reduce the activity coefficients of salt ions in the soil liquid phase system. Among them, the application concentrations of 5% and 10% OSiFs showed significant antagonistic effects on ions such as Na^+^ and Cl^−^ and effectively regulated the soil pH to a suitable range for crops (6.8–7.2). Through X-ray photoelectron spectroscopy (XPS) characterization technology, it was found that the organic silicon components reacted with the Lewis basic sites on the soil colloids, resulting in a significant characteristic shift in the binding energy of the Si 2p orbitals. This provided experimental evidence for explaining the molecular mechanism by which organic silicon materials alleviate salt stress through chemical bonding pathways. Based on the above findings, this study constructed a combined application mode of OSiFs and traditional inorganic silicon fertilizers. It was verified that this mode could reduce the sodium adsorption ratio (SAR) of saline–alkali soil and enhance the root vitality of crops, preliminarily achieving the biological restoration and improvement of soil fertility in saline–alkali barrier farmland. Based on the conclusions of this study, we propose the following forward-looking suggestions: The combined application mode of organic silicon fertilizers (OSiFs) and inorganic silicon fertilizers should be regarded as a sustainable agricultural and saline–alkali soil improvement strategy. To promote the practical application and environmental safety assessment of this strategy, subsequent research should focus on the following aspects: 1. long-term field trials and environmental fate assessment; 2. research on ecosystem cascade effects and biological safety; 3. the formulation of precise application norms based on mechanisms.

## Figures and Tables

**Table 1:** Soil moisture content, total salt content, electrical conductivity, and pH value after infiltration.

Sample Name	Total Salt Content g/kg	Soil Moisture Content (*w*/*w*, %)	Soil Conductivity	Soil pH
Y upper	0.95	34.67%	86.75	9.91
Y middle	5.75	31.46%	171.9	9.89
Y lower	7.9	37.41%	609	9.53
Y+F upper	1.52	31.42%	136.36	9.21
Y+F middle	8.32	33.83%	214.2	10.14
Y+F lower	5.03	33.72%	453	10.03
Y+5% upper	0.95	33.14%	88.36	9.6
Y+5% middle	6.95	33.42%	204.56	10.19
Y+5% lower	3.9	35.95%	463.33	10.05
Y+10% upper	1.33	31.76%	109.46	9.53
Y+10% middle	6.9	33.13%	191.36	10.08
Y+10% lower	4.88	28.63%	439.66	9.91

## Data Availability

The original contributions presented in this study are included in the article/Supplementary Material. Further inquiries can be directed to the corresponding authors.

## Supplementary Materials

The following supporting information can be downloaded at https://www.mdpi.com/article/10.3390/polym18020231/s1. Figure S1: Hydrogen spectrum of organosilicon macromolecules; Figure S2: Py-GCMS test spectra of organosilicon molecules; Figure S3: Thermogravimetric analysis of organosilicon molecules; Table S1: Electron binding energy of O1s in modified silicone before and after.
