# Supplementary material for: Effects and Mechanisms of Silicone Fertilizer on Salt Ion Activity in Saline–Alkaline Soils
Source: Polymers (Basel). 2026 Jan 16;18(2):231. doi: 10.3390/polym18020231 (PMC12845716; doi:10.3390/polym18020231)
Supplement: Supplementary file 1 [file polymers-18-00231-s001.zip › polymers-3956880-supplementary.pdf]

Supplementary materials

Effects and Mechanisms of Silicone Fertilizer on Salt Ion Activity in Saline-Alkaline Soils

Furu Song [a], Dongxia Li [c], Liqiang Song [a], Ziku Cao [d], Zhipei Cao [e], Yafei Sang [b]*, Lianwei Kang [b]*

[a] Hebei Silicon Valley Fertilizer Co., Ltd, Handan 056038, Hebei, China

[b] College of Materials Science and Engineering, Hebei University of Engineering, Handan, 056038, China

[c] School of Water Conservancy and Hydropower, Hebei University of Engineering, Handan, Hebei 056038, China

[d] Soil and Fertilizer Station of the Agricultural and Rural Affairs Bureau of Yongnian District, Handan City, Handan 056038, Hebei, China

[e] Hebei Silicon Valley Agricultural Science Research Institute, Handan 056038, Hebei, China

1. Supporting experiment section

Figure S1 Hydrogen spectrum of organosilicon macromolecules

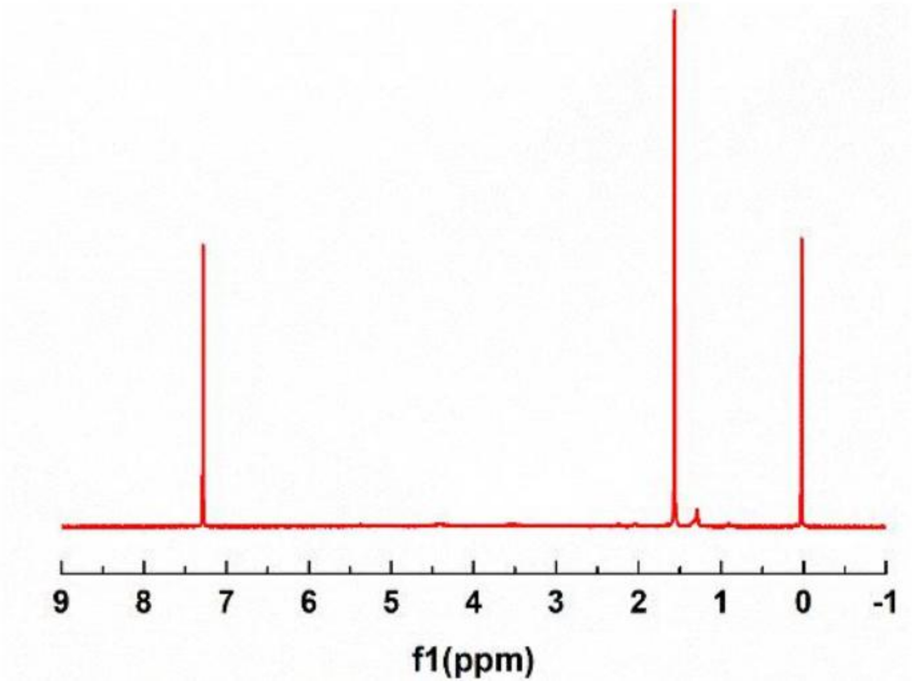

Figure S2 Py-GCMS test spectra of organosilicon molecules

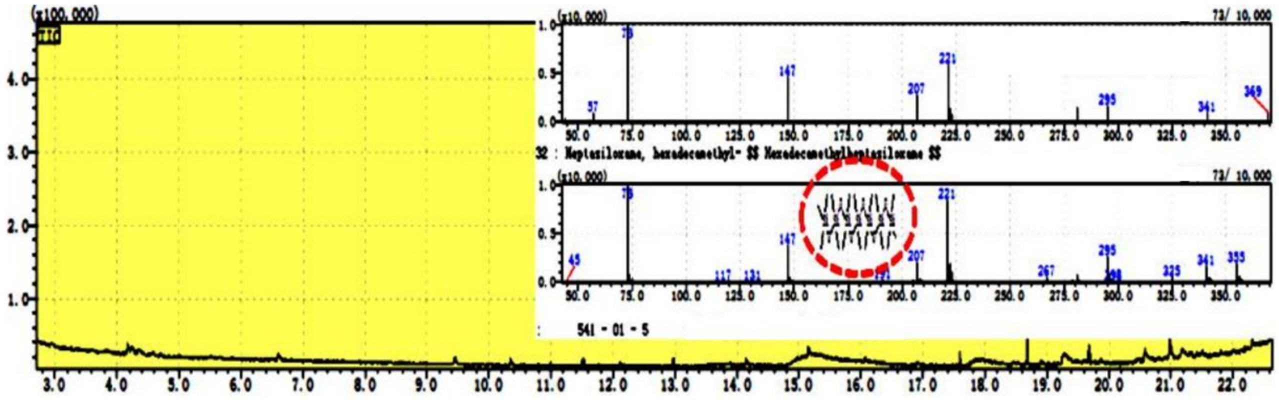

Figure S3 Thermogravimetric analysis of organosilicon molecules

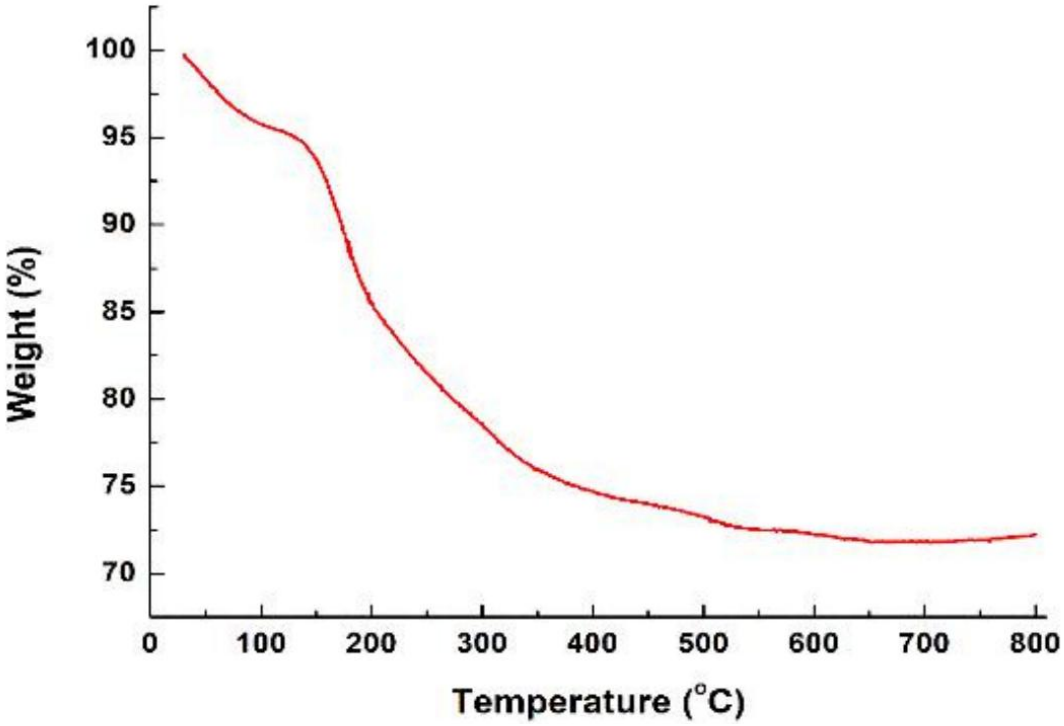

Table S1 Electron binding energy of O1s in modified silicone before and after reaction

	Modified Organosilicon O1s Peak BE	Sample+Modified Organosilicon O1s Peak BE
Al_2O_3	532.74	532.67
$ZnCl_2$	532.74	532.63
$CuSO_4$	532.74	532.57
